# Supplementary material for: Introducing a translationally relevant mouse model of radiosurgery-induced unilateral hearing loss
Source: Front Neurosci. 2026 May 7;20:1748309. doi: 10.3389/fnins.2026.1748309 (PMC13189964; doi:10.3389/fnins.2026.1748309)
Supplement: Supplementary file 1 [file Data_sheet_1.pdf]

Supplementary figures

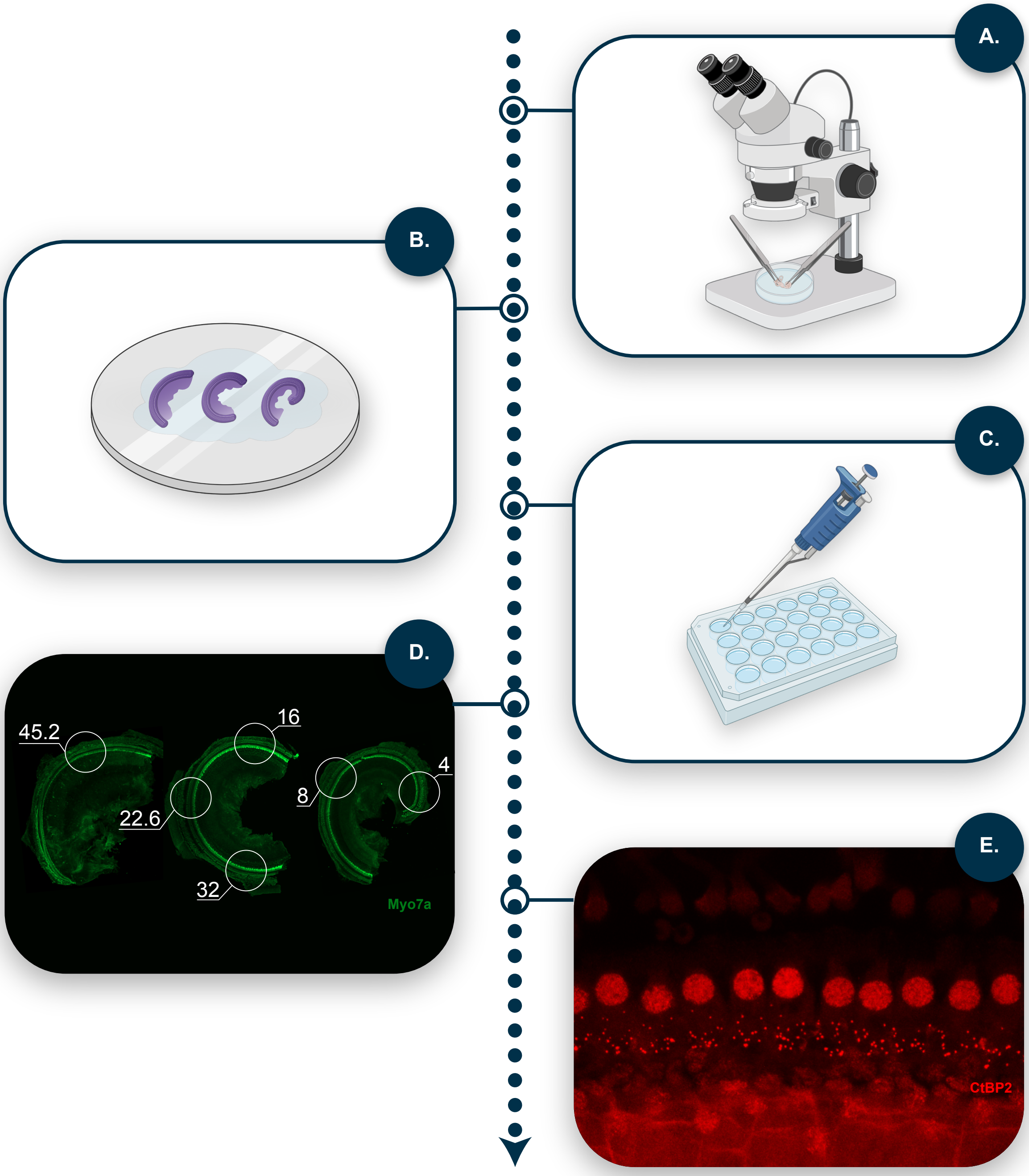

Supplementary Fig. S1 Cochlear dissection, whole-mount preparation, and frequency-specific quantification workflow.
(A) Dissection of the decalcified cochlea and segmentation of the cochlear sensory epithelium into apical, middle, and basal turns under a stereomicroscope.
(B) Adhesion of cochlear segments onto 12 mm round coverslips using Cell-Tak tissue adhesive.

(C) Coverslips with samples were placed in PBS in 24-well plates and processed for immunostaining.
(D) Representative cytocochleogram showing cochlear frequency mapping generated using the Measure_Line plugin from the Eaton-Peabody Laboratories Histology Core (Mass Eye and Ear, https://masseyeandear.org/research/otolaryngology/eaton-peabody-laboratories/histology-core), enabling quantification of hair cell survival in 300 µm-long segments at defined frequency regions.
(E) The previously generated tonotopic map was used as a guide to acquire high-magnification confocal images of the inner hair cell region, within 10–15 inner hair cells at each frequency region.
Panels A–C include elements created with BioRender.com.

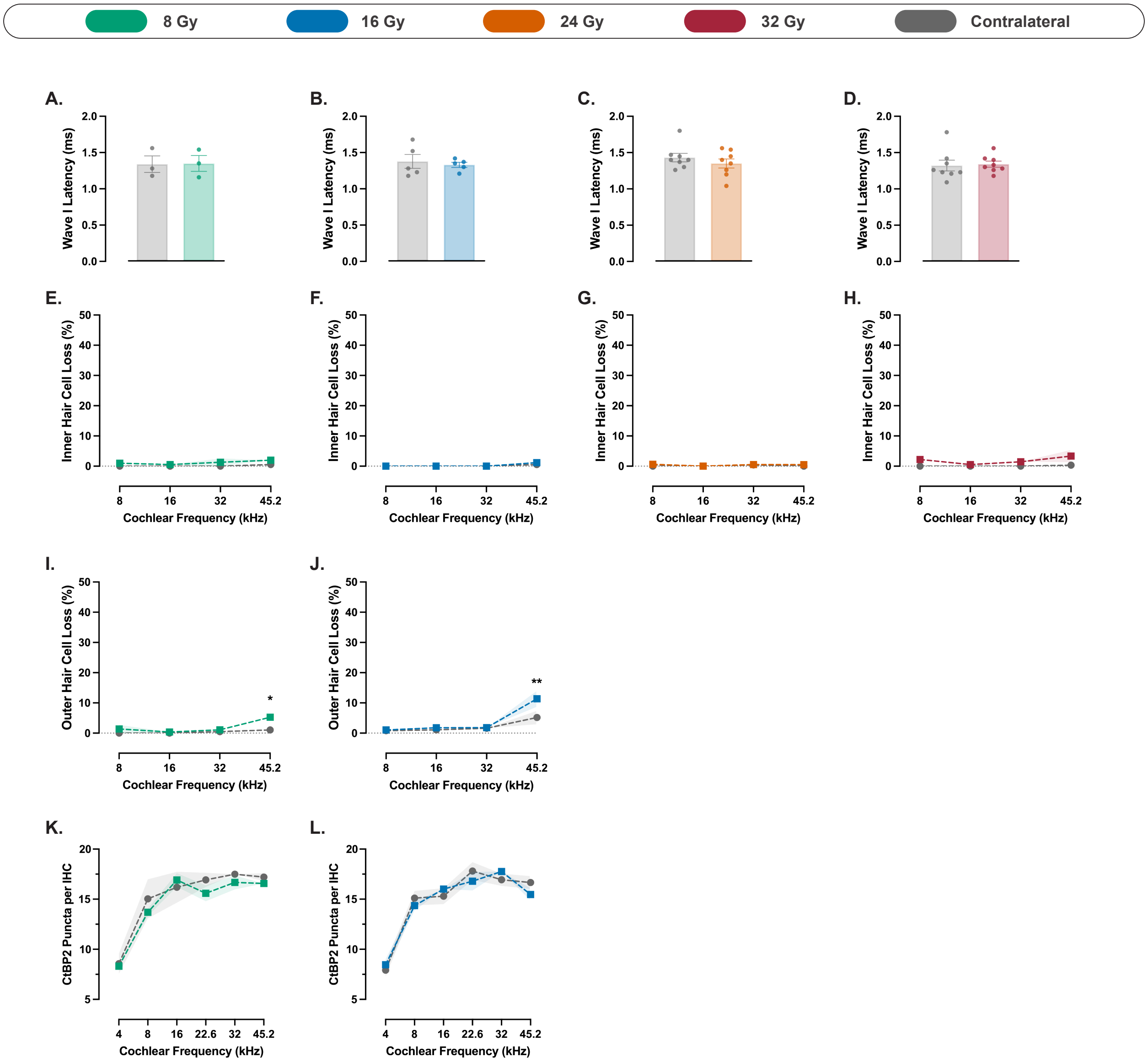

Supplementary Fig. S2 Additional auditory and histological outcomes across radiation dose groups.

(A–D) Wave I latency at week 4 following cochlear irradiation with 8 Gy (A; n = 3), 16 Gy (B; n = 5), 24 Gy (C; n = 8), and 32 Gy (D; n = 8), showing no significant interaural differences.

(E–H) Inner hair cell (IHC) loss across cochlear frequencies at week 4 in the 8 Gy (E; n = 3), 16 Gy (F; n = 5), 24 Gy (G; n = 5), and 32 Gy (H; n = 5) groups, with no significant differences observed between irradiated and contralateral ears.

(I–J) Percentage outer hair cell (OHC) loss across cochlear frequencies in mice exposed to 8 Gy (I; n = 3) and 16 Gy (J; n = 5), relative to the contralateral cochlea, showing mild but significant interaural differences at 45.2 kHz.

(K–L) Ribbon synapse density measured as CtBP2-positive puncta per inner hair cell in the 8 Gy (K; n = 3) and 16 Gy (L; n = 5) groups, showing no significant differences between irradiated and contralateral cochlea.

Data are presented as means with error bars or shaded areas representing SEM. Asterisks indicate statistical significance (*p < 0.05, **p < 0.01). Statistical analysis was performed using two-way repeated measures ANOVA with Bonferroni correction for *post hoc* pairwise comparisons. Colors correspond to radiation dose groups as shown in the legend. The contralateral ear serves as control in all cases.
